# Comparison of the effectiveness of two piperazine based nano-catalysts in the synthesis of benzoxazoles and benzimidazoles and use of the powerful one in the *N*-Boc protection of amines†

**DOI:** 10.1039/d4ra01300j

**Published:** 2024-03-19

**Authors:** Maryam Mousapour, Farhad Shirini

**Affiliations:** a Department of Chemistry, College of Sciences, University of Guilan Post Box: 1914 Rasht 41335-19141 Iran shirini@guilan.ac.ir +98 131 3233262 +98 131 3233262

## Abstract

In this work, a comparison between the catalytic activity of two piperazine based ionic liquids immobilized on ZnO NPs and SiO_2_ NPs is presented in the synthesis of benzoxazoles and benzimidazoles. These reactions are performed under solvent free conditions during appropriate reaction times with high yields. The catalyst obtained from ZnO-NPs (PINZS), as the more efficient one, is used for the efficient promotion of the *N*-Boc protection of amines. High chemoselectivity, no by-products, facile separation of the catalyst, short reaction times and no need for volatile organic solvents are the best features of the proposed methods.

## Abstract 2

In this work, a comparison between the catalytic activity of two piperazine based ionic liquids immobilized on ZnO NPs and SiO_2_ NPs is presented in the synthesis of benzoxazoles and benzimidazoles.

## Graphical abstract

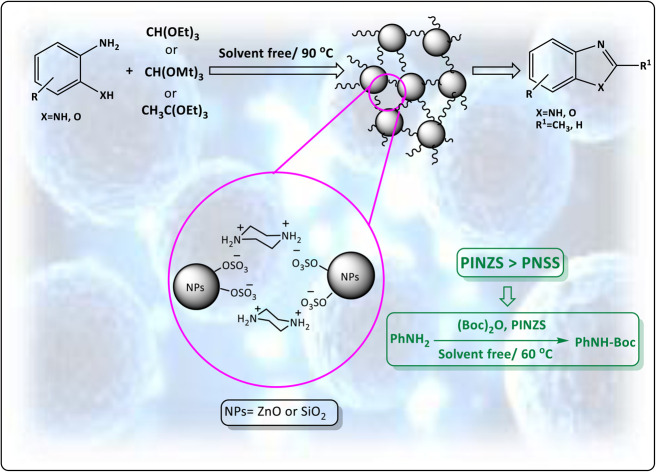

## Introduction

Nanoparticles with sizes between 10 and 500 nm can be observed widely in nature and have capacious usage in many sciences,^1^ from synthetic processes (chemistry) and industrial activity to treatments for diverse diseases (medicine),^2^ physics,^3^ geology^4^ and so on. Nanoparticles (NPs) often represent phenomena that are not seen at other scales because of their transmission between bulk and atomic structure of materials. In fact, the small size of them leads to a lower concentration of point defects compared to their bulk ones. NPs and their industrialized derivatives including dyes, ceramics, plastics and magnetic products are very significant reagents of atmospheric pollution.^5^ Among these reagents, nano-sized metal oxides became very beneficial candidates for the preparation of efficient catalysts for industrial applications due to their high hydrothermal stability, ease of handling, large surface area, cheapness, and non-corrosiveness.^6–9^

ZnO NPs are a well-known example of nano-sized metal oxides in industry and science with selective toxicity to bacteria with minimal influence on animal and human cells. Zinc oxide nanoparticles based on their biocompatibility have been utilized widely for preserving agricultural products and foods and as drug carriers.^10^ Using ZnO NPs as a heterogeneous catalyst has attracted a lot of attention in chemistry, especially in the organic field, due to high catalytic performance, easy work-up process and ability to be recovered, to be economical and simplicity of isolation. They have been used in organic reactions as solid supports for the immobilization of ionic liquids.^11–14^

Similarly, nanostructured silica or silicon dioxide nanoparticles are other nanoparticles that have drawn great attention because of their high stability, low toxicity and potential to be functionalized with a range of substrates such as molecules and polymers. These nanostructured reagents with notable properties can make improvements in biomaterial conjugates with different hybrid nanomaterials.^15^ SiO_2_ NPs due to their large surface area, nanometer size, high hydrothermal stability and easy handling became very beneficial materials for the preparation of heterogeneous nanocatalysts leading to considerable industrial applications.^16^

The catalytic performance of nanoparticles is affected by their size and their reactivity and selectivity pertained on the various crystallographic planes on NPs, which can be gained by controlling the morphology of them.^17^ Nanocatalysts are utilized in wide range of organic transformations^18,19^ and various applications which can be prepared by chemical vapor synthesis, sol–gel technique, chemical precipitation, photochemical method, hydrothermal method, antisolvent precipitation, glow discharge plasma electrolysis, wet-chemical method, microwave irradiation, and sonochemical method, thermal decomposition and microarc oxidation irradiation.^20–25^

Although, in last years, ionic liquids (ILs) with their substantial properties can be specific choices in different areas of chemistry (*e.g.*, organic and inorganic chemistry, electrochemistry, and so on),^26^ difficulty in separation and large amount utilization of them, caused the limitation of their usage in industry and synthetic chemistry. In the today's chemistry world, immobilization of ionic liquids on solid supports is considered as an impressive procedure for the conversion of ILs to their heterogeneous homologues, leading to their stability and recoverability.^27^ This protocol incorporates the advantages of the heterogeneous catalysts along with homogeneous ionic liquid layers in one reagent system. In this area, modification of the selected supports can lead to the air and moisture-resistant catalysts with noncorrosive nature, which can be simply separated from the reaction media. This feature in according with the green chemistry rules decreases the contamination and wastage of materials. Organic polymers, clays, metal oxides and zeolites are commonly used as supports in immobilization of ionic liquids.^28–31^

**Scheme 1:**
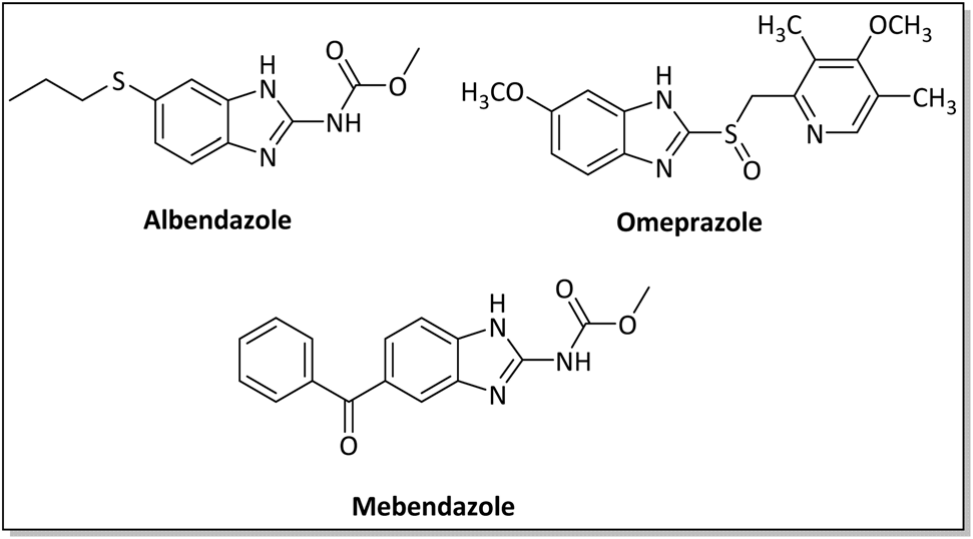
Commercial drugs containing benzimidazole ring.

Benzimidazoles, benzoxazoles and their derivatives are an important group of heterocyclic compounds that have significant biological properties such as antitumor,^32^ anti-inflammatory,^33^ antimicrobial^34,35^ and anticancer^36^ activities. They are main intermediates for the synthesis of some drugs, for example, there is a benzimidazole framework in the structure of vitamin B12. Also, these derivatives are used as important intermediates for the synthesis of various drugs such as albendazole (inhibiting intestinal infection in AIDS patients), omeprazole (proton pump inhibitor) and mebendazole (Scheme 1).^33,35^ Moreover, these heterocyclic compounds can be widely used as ligands for transition metals.^37,38^ Therefore, some methods and catalysts have been reported for the synthesis of these types of biological compounds, but there are disadvantages and problems such as long reaction times, use of a large amount of catalyst, the need for volatile organic solvents and the impossibility of recovering the catalyst caused the trend towards efficient methods and catalysts to overcome these problems.

Protection of amines in synthetic organic chemistry has countless applications, especially for the synthesis of complex natural products. The synthesis of peptides, which are the most biologically important molecules, is of great importance. It is not possible to make a peptide with a specific structure from its amino acids unless the amine groups are properly protected to control the coupling reaction and polymerization.^39^ In addition, protected amines have found many applications in different areas. Different types of methods can be used for the protection of amine group, which of them acylation, formylation, alkylation, benzylation, carbobenzyloxylation and *N*-Boc protection are the most important ones. For example, the *N*-Boc protection of amines is a key technique in medicine synthesis such as synthesis of molecules for COVID-19 treatment.^40^ Several methods with different reaction conditions have been reported to accelerate the protection of the NH_2_ group by these protecting agents which despite their advantages, have weaknesses such as long reaction times, low yields, use of organic solvents. The reported catalysts suffer from tedious, costly and non-recyclable separation methods.^41–44^ Therefore, the use of new and efficient catalytic systems, which can solve the mentioned problems as much as possible, is one of the main challenges.

After extensive studies on the introduction and application of various catalysts in our research group,^45–51^ and especially the use of nano-catalysts such as piperazine immobilized on nano-ZnO-sulfuric acid (PINZS) and piperazinium nano silica sulfonate (PNSS) in the synthesis of some organic compounds,^52,53^ in this research we decided to compare the catalytic properties of PINZS and PNSS in the synthesis of benzimidazole and benzoxazole derivatives. This research shows the importance of choosing of the appropriate support in the immobilization of ionic liquids. In continue, the more forceful catalyst was used in the *N-tert*-butoxycarbonylation of amines, in order to eliminate all or some of the limitations accompanied with the previously reported methods used for the same reactions.

## Experimental

All chemicals which used in this study such as di-*tert*-butyldicarbonate, amines, orthoesters, solvents *etc.* were purchased from Merck (Germany), Aldrich (America) and Fluka (Switzerland) chemical companies with no need of further purification. Characterization of the obtained products was determined using melting point, FT-IR, and NMR spectra of them compared with those reported in the literature. Thin-layer chromatography (TLC) method applying silica-gel SIL G/UV 254 plates was carried out for the reaction monitoring and purity determination of the substrates.

### Preparation of the catalysts (PINZS and PNSS)

At first and in order to synthesis nano ZnO-SO_3_H and or SiO_2_-SO_3_H which reported in our previous studies, ClSO_3_H (1 mL) was reacted with nano-ZnO and or nano-SiO_2_ (1 g) in dry dichloromethane (CH_2_Cl_2_) (25 mL) in dropwise method over a period of 15 min at 0 °C (using an ice bath). In order to send the produced HCl in to an absorbing solvent (H_2_O), a gas outlet tube was used. After completion of acid addition, the reaction mixture was additionally stirred at room temperature (12 h). After that, the residue was filtered and washed with diethyl ether (2 × 10 mL). After drying at 80 °C for 1 h, nano ZnO-SO_3_H and or SiO_2_-SO_3_H were achieved as the white powder.

**Scheme 2:**
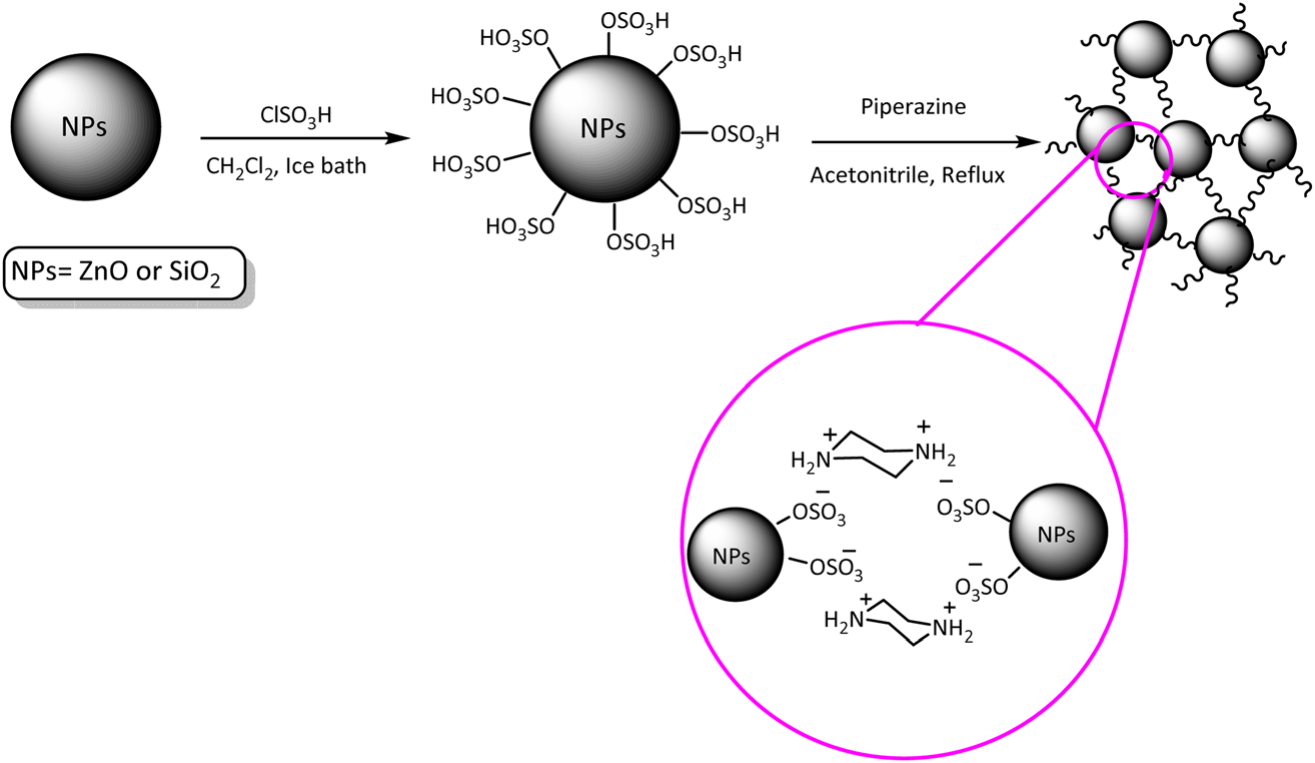
Preparation of PINZS and PNSS.

In the next step, in a round-bottomed flask, piperazine (130 mg, 1.5 mmol) was added to the prepared nano particles (1.3 g) in CH_3_CN (25 mL) and the reaction mixture was stirred at reflux temperature for one day (then, in the case of PNSS, the resulting slurry was centrifuged). In continue, the obtained solid was filtered and washed with CH_3_CN to remove the extra piperazine. After drying for 1 h (80 °C) PNSS (as opalescent powder (1.4 g)) and PINZS (as white powder (1.4 g)) were obtained (Scheme 2).^52,53^

### General procedure for the synthesis of benzoxazole and benzimidazole

In a round-bottom flask (10 mL), a mixture of amine (1 mmol), orthoester/formic acid (1/5 mmol) and PINZS/PNSS (10 mg) was stirred and heated at 90 °C in an oil-bath for the appropriate time. When the reaction was fulfilled [monitored by TLC: *n*-hexane : ethyl acetate (7 : 3)] the reaction mixture was cooled to room temperature, ethyl acetate (20 mL) was added to it and the catalyst was separated by filtration. Water and Na_2_SO_4_ were used for washing of the organic phase and its drying, respectively. In continue evaporation of the solvent, gave the favorable product in good to high yields.

### General procedure for *N-tert*-butoxycarbonylation of amines

In a round-bottom flask (10 mL), a mixture of amine (1 mmol), di-*tert*-butyldicarbonate [(Boc)_2_O] (2 mmol) and PINZS (20 mg) was stirred and heated at 60 °C in an oil-bath for the appropriate time. When the reaction was completed [monitored by TLC: *n*-hexane : ethyl acetate (7 : 3)] the reaction mixture was cooled to room temperature, ethyl acetate (20 mL) was added to it and the catalyst was separated by filtration. Water and Na_2_SO_4_ were used for washing of the organic phase and its drying, respectively. Evaporation of the solvent, gave the requested target product in good to high yields. Spectral data (FT-IR, ^1^H NMR and ^13^C NMR) for new compound is represented below.

#### Di-*tert*-butyl(sulfonylbis(1,3-phenylene))dicarbamate

FT-IR (KBr): *v* = 3333, 2981, 1730, 1700, 1595, 1533, 1480 cm^−1^. ^1^H NMR (500 MHz, DMSO-d_6_) *σ*: 1.59 (9H, s, 3 × CH_3_), 7.58–7.64 (2H, m, arom), 7.74 (1H, d, *J* = 8 Hz, arom), 8.28 (1H, s, arom), 9.92 (1H, s, NH) ^13^C NMR (100 MHz, DMSO-d_6_): 27.97, 79.80, 115.79, 120.55, 122.59, 130.18, 140.67, 141.48, 152.58.

## Result and discussion

### Catalytic activity

**Scheme 3:**
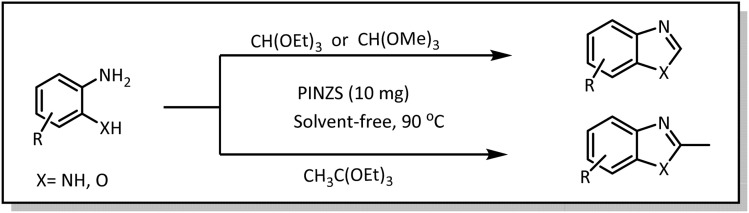
Preparation of the benzoxazole and benzimidazole derivatives under the optimized reaction conditions in the presence of PINZS.

At the first step of this study and in order to determine the best reaction conditions the interaction of 1,2 phenylene diamine and triethyl orthoformate was selected as the model one and the effect of various amounts of PINZS and different temperatures was studied on it. The reaction was also performed in H_2_O, EtOH, CH_3_CN and also under solvent free conditions. The results presented in Table 1 show that the best results can be obtained using 10 mg of PINZS and 1.5 mmol of triethyl orthoformate in 90 °C in the absence of solvent. There is no more improvement in aspect of times and yields under the other conditions (Scheme 3).

**Table 1:** Optimization of the reaction conditions for the synthesis of benzimidazole catalyzed by PINZS

Entry	Cat. (mg)	Triethyl orthoformate (mmol)	Temperature (°C)	Solvent	Time (min)	Yield^a^ (%)
1	40	1/5	60	—	17	98
2	30	1/5	60	—	60	96
3	20	1/5	60	—	25	95
4	10	1/5	60	—	20	98
5	10	1/5	90	—	10	98
6	10	1	90	—	30	94
7	10	2	90	—	9	96
8	10	1/5	Reflux	H_2_O	60	40
9	10	1/5	Reflux	EtOH	60	50
10	10	1/5	Reflux	CH_3_CN	60	30

a Isolated yield.

In continue, use of different orthoesters (including trimethyl orthoformate, triethyl orthoformate, and triethyl orthoacetate) and also formic acid in the same optimized reaction condition resulted a variety of benzimidazole and benzoxazole derivatives in the presence of PINZS. As can be seen in Table 2, all compounds were obtained in high yields and good times in both methods (Table 2, entries 1–12). The results also show that 1,2-phenylene diamines containing electron-withdrawing groups gave the target products in excellent yields (Table 2, entries 4). Whereas 1,2-phenylene diamines with electron-donating groups reacted at longer reaction times (Table 2, entries 5–8). Benzoxazole derivatives were also obtained in the presence of this catalyst (Table 2, entries 9–12). Our studies also clarified that the same reactions can be applied for the synthesis of the mentioned products using PNSS instead of PINZS. Comparison between the catalytic activities of PINZS and PNSS clearly showed that the reactions were performed in shorter reaction times in the presence of PINZS than PNSS. This indicates that although the existence of ionic liquid is an important factor to speed the reaction up but the nature of the surface used to immobilize the ionic liquid is also essential to accelerate the reaction. This observation can be due to the more Lewis acidic characteristics of ZnO NPs.

**Table 2:** Synthesis of benzimidazole and benzoxazole derivatives catalyzed by PNSS and PINZS as nano catalysts

Entry	Amine	Orthoester/formic acid	Product	Time (min)			M. P. (°C)	
				Yield^a^ (%)			Observed	Reported
				ZnO		SiO_2_		
1	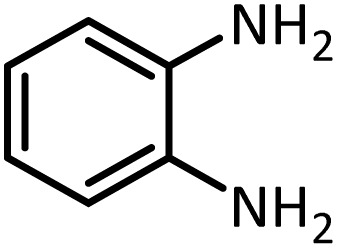	CH(OEt)_3_	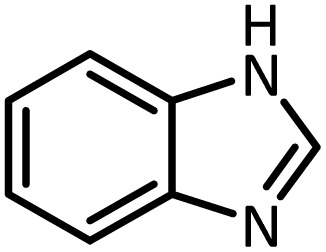	10		25	164–166	168–170 (ref. 54)
				98		96		
2	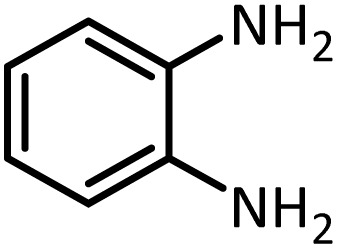	CH(OMe)_3_	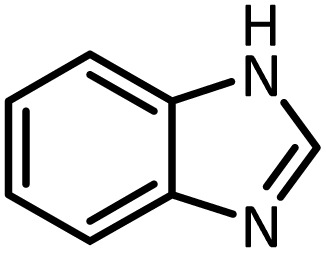	15		17	164–166	168–170 (ref. 54)
				94		93		
3	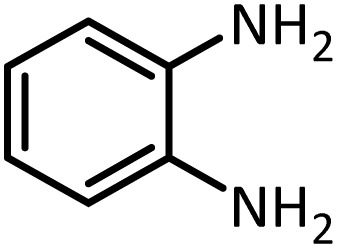	HCOOH	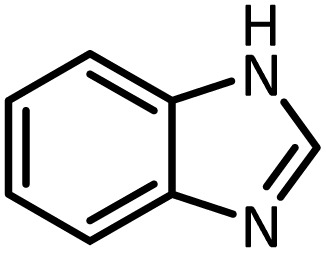	10		13	164–166	168–170 (ref. 54)
				92		92		
4	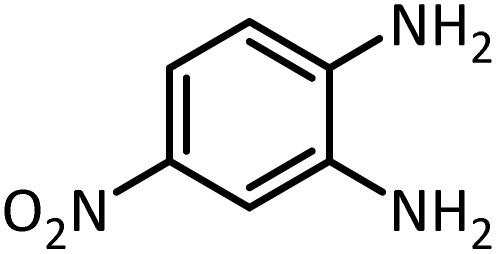	CH(OEt)_3_	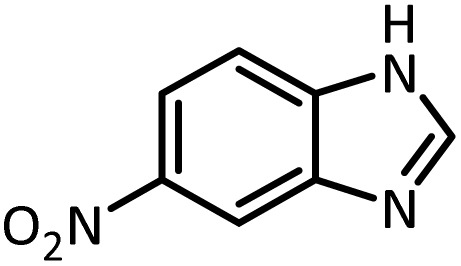	13	20		198–200	203–205 (ref. 54)
				96	70			
5	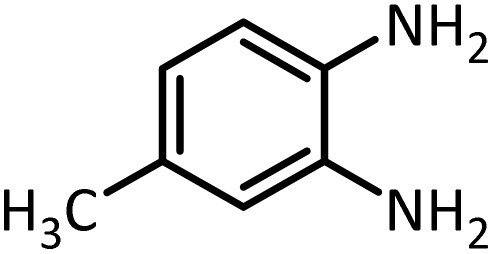	CH(OEt)_3_	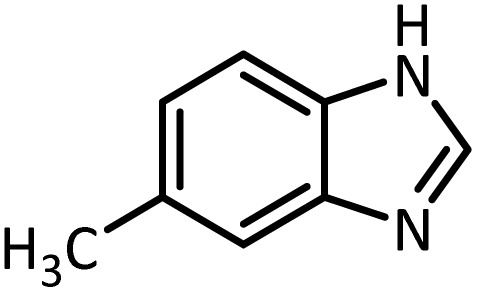	12	30		107–109	109–111 (ref. 54)
				93	97			
6	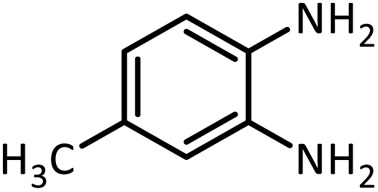	CH(OMe)_3_	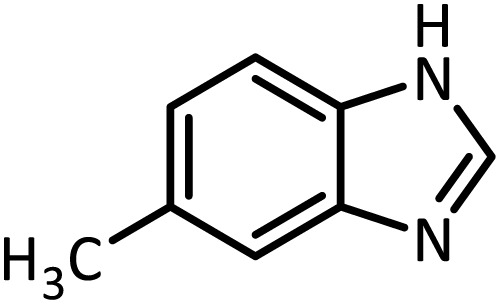	45	60		107–109	109–111 (ref. 54)
				95	94			
7	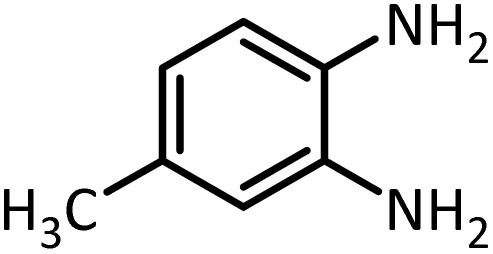	CH_3_CH(OEt)_3_	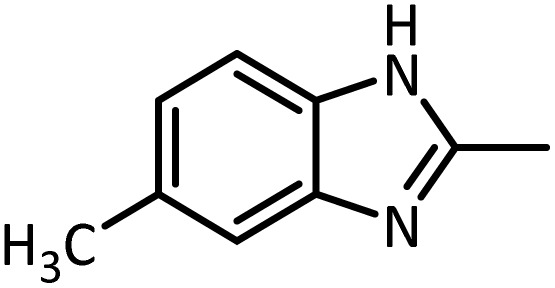	30	50		194–196	194–196 (ref. 54)
				92	90			
8	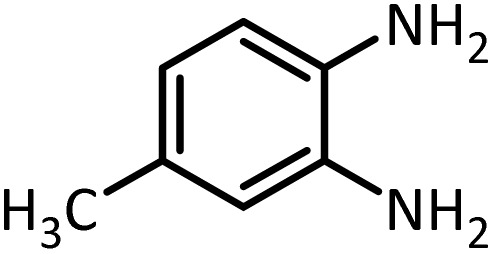	HCOOH	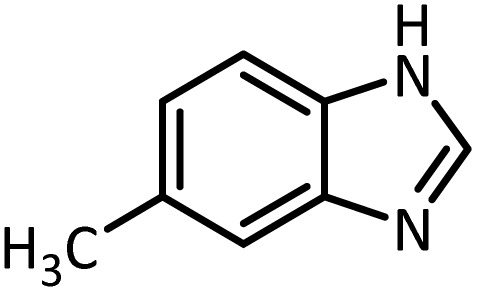	35	40		107–109	109–111 (ref. 54)
				98	97			
9	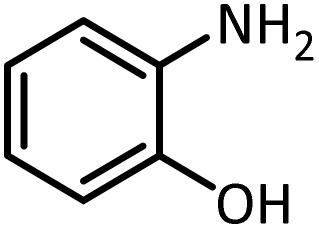	CH(OEt)_3_	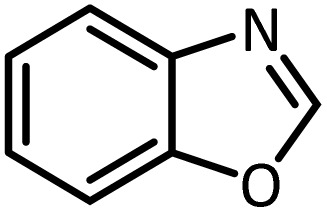	7	15		Oil	Oil^54^
				96	94			
10	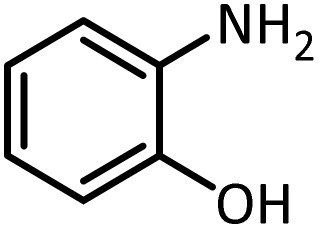	CH(OMe)_3_	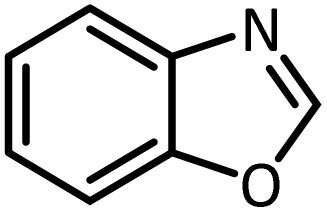	5	5		Oil	Oil^54^
				96	92			
11	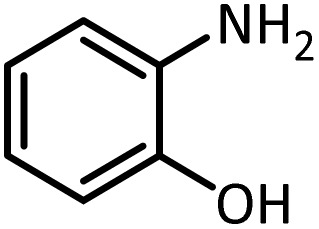	CH_3_CH(OEt)_3_	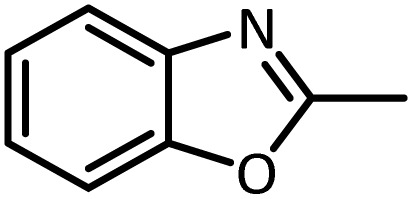	3	5		Oil	Oil^54^
				98	96			
12	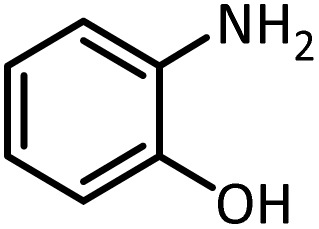	HCOOH	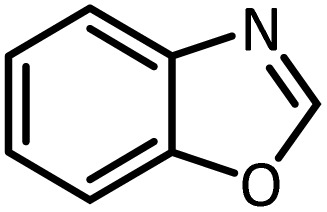	12	20		Oil	Oil^54^
				94	93			

a Isolated yield.

Considering the ability of PINZS to accelerate the synthesis of benzimidazoles and benzoxazoles, it was decided to investigate the potential of its use in other organic transformations such as protection reactions. For this purpose, *N*-Boc protection of amines was also studied in the presence of PINZS catalyst.

Optimization of the reaction conditions for the synthesis of *N-tert*-butoxycarbonyls of amines was done by studying the reaction of aniline (1 mmol) with di-*tert*-butoxypyrocarbonate [(Boc)_2_O] (2 mmol) in the presence of various amounts of PINZS at a range of 25 to 90 °C under the solvent less conditions. This reaction was also performed in different solvents such as CH_2_Cl_2_, H_2_O and EtOH under thermal conditions.

**Scheme 4:**
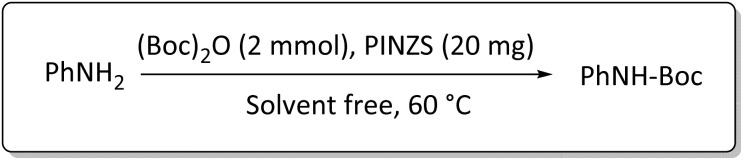
Best conditions for the *N*-Boc protection of amines in the presence of PINZS.

The obtained results clarified that 20 mg of the catalyst was adequate to promote the reaction effectively at 60 °C and under solvent-free conditions (Table 3, entry 5) (Scheme 4). Any further improvement in terms of the reaction times and yields was not observed under the other conditions. These results also showed that the reaction was not proceeded considerably in the absence of the catalyst even after prolonged reaction times (Table 3, entry 1). Therefore, PINZS is necessary to proceed the reaction.

**Table 3:** Optimization of the reaction conditions for the synthesis of *N-tert*-butoxycarbonylation of amines catalyzed by PINZS

Entry	Cat. (mg)	Solvent	Temperature (°C)	Time (min)	Yield^a^ (%)
1	40	—	r.t.	1 h	50
2	40	—	60	1 h	98
3	40	—	90	1 h	90
4	60	—	60	50	97
5	20	—	60	15	93
6	10	—	60	20	95
7	20	—	60	35	90
8	20	—	r.t.	50	91
9	20	Water	60	25	88
10	20	CH_2_Cl_2_	Reflux	1 h	20
11	20	EtOH	Reflux	1 h	70

a Isolated yields.

In order to show the efficiency of the best conditions, a broad range of amines, including aromatic, heterocyclic, heteroaromatic and aliphatic ones were protected using this method and the consequences were categorized in Table 4.

**Table 4:** Protection of amines catalyzed by PINZS

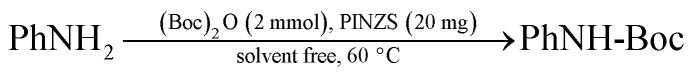				
Entry	Amine	Product	Time (min)	Yield^a^ (%)
1	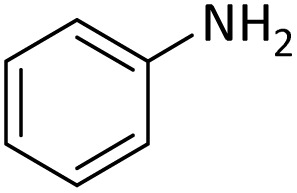	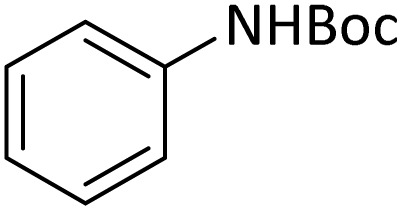	5	95
2	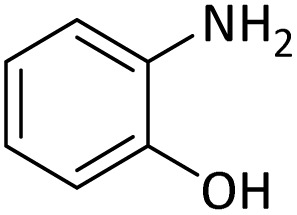	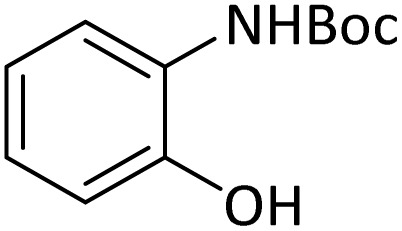	4	93
3	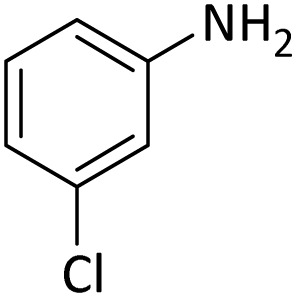	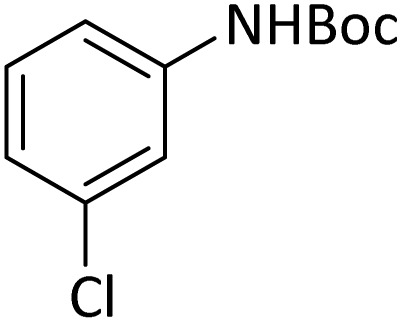	30	91
4	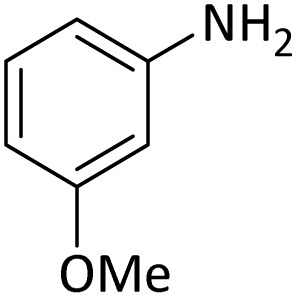	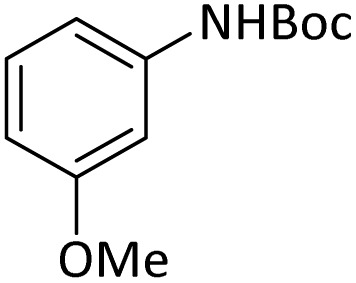	10	95
5	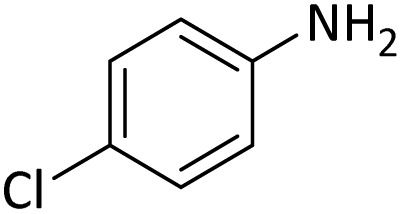	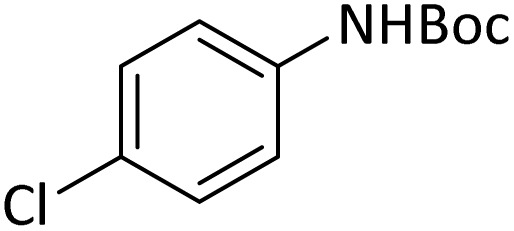	15	94
6	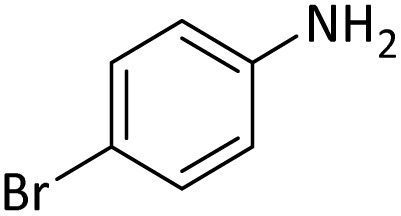	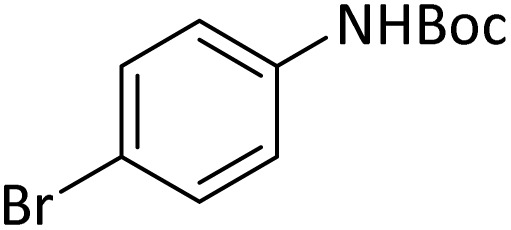	4	92
7	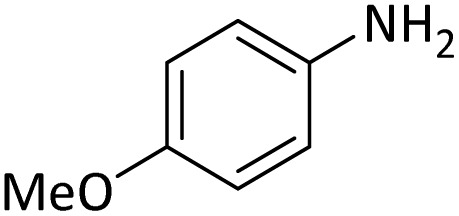	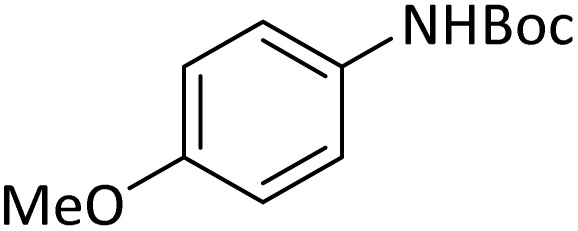	3	94
8	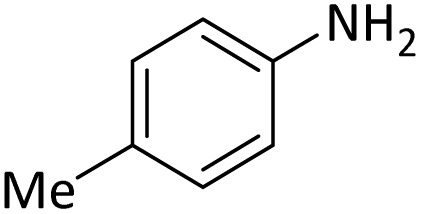	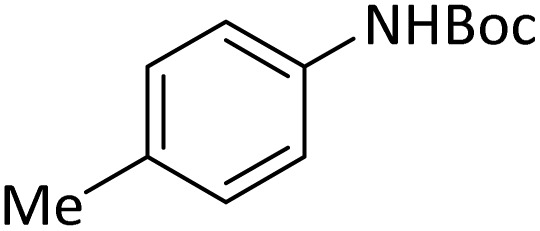	8	96
9	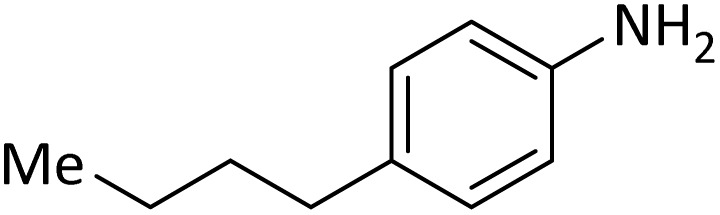	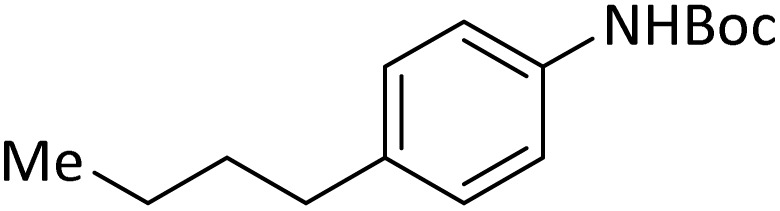	15	95
10	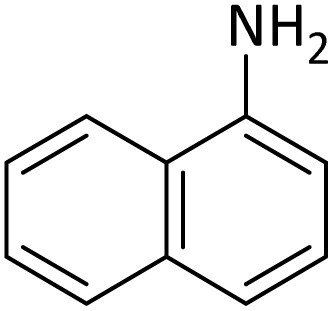	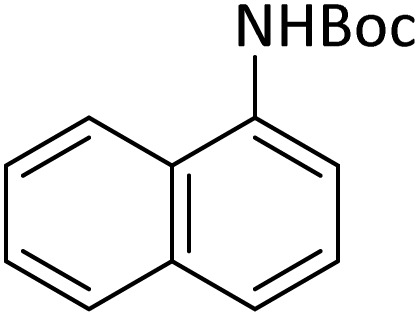	25	91
11	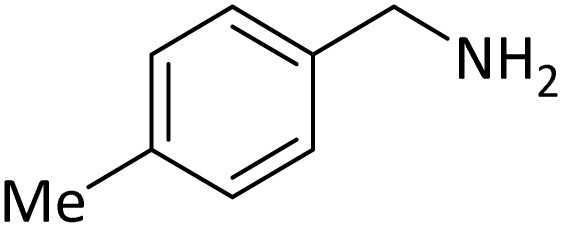	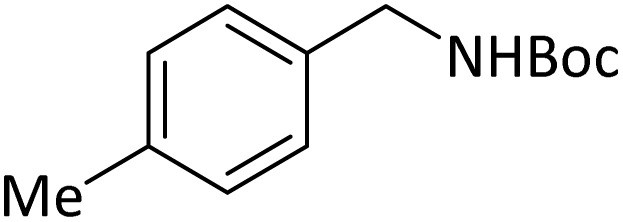	10	90
12	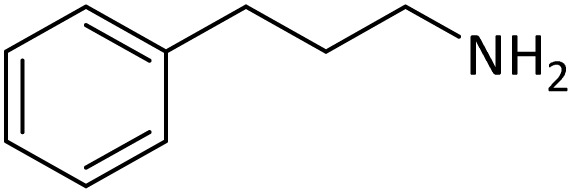	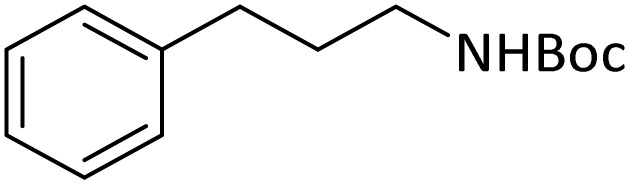	10	91
13	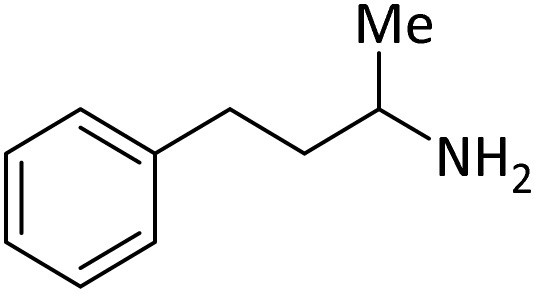	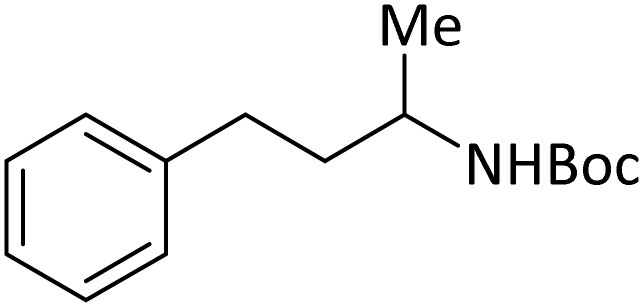	5	95
14	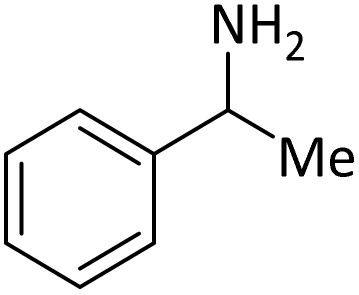	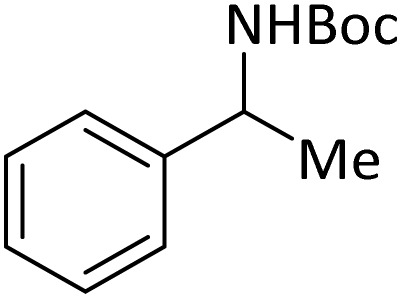	12	96
15	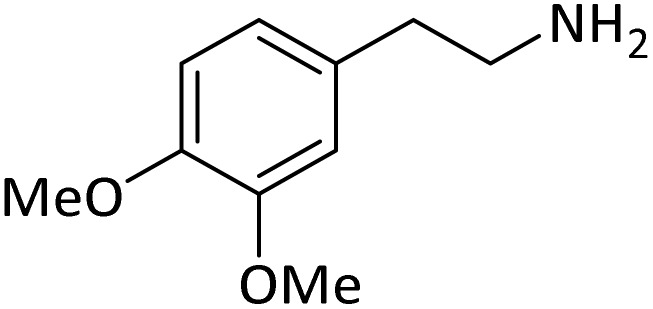	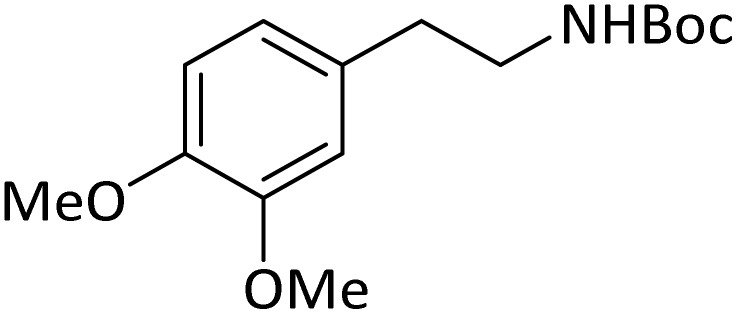	3	93
16	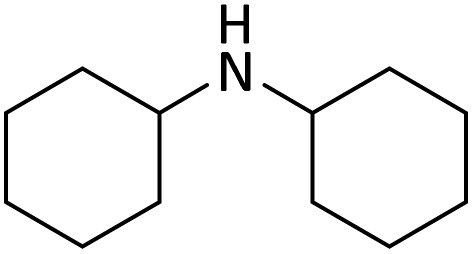	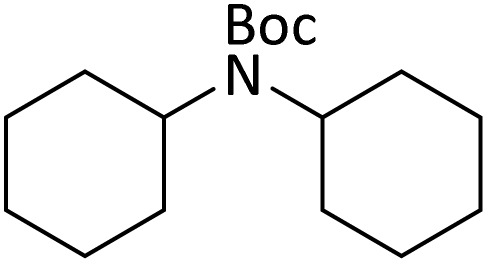	15	92
17	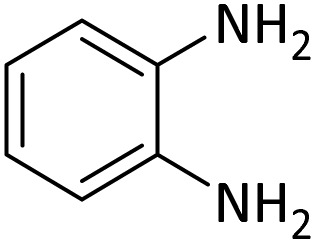	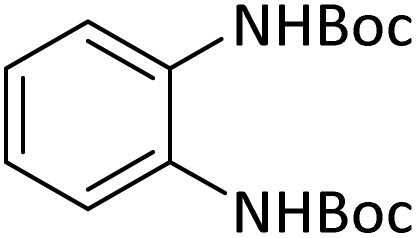	4	98^b^
18	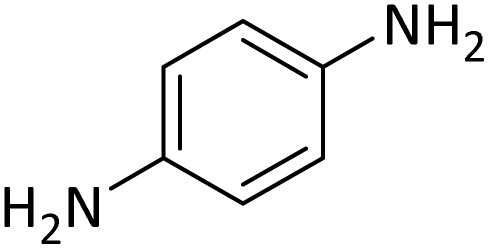	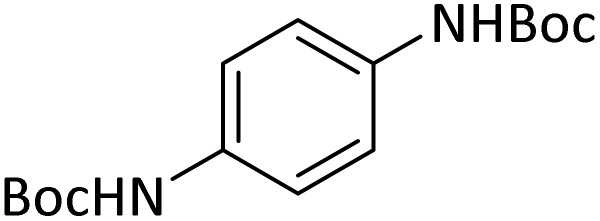	6	98^b^
19	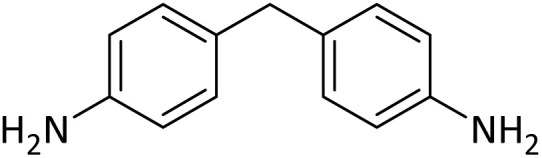	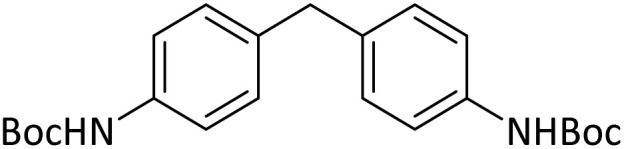	15	90^b^
20	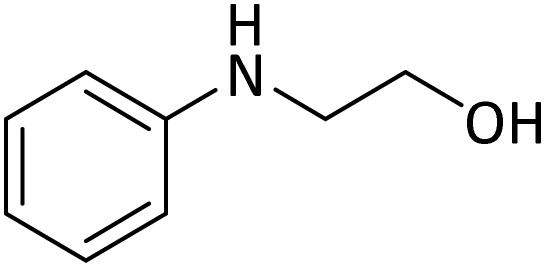	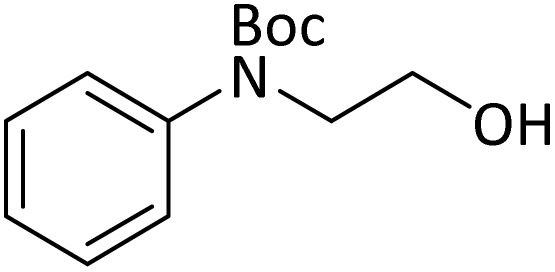	5	92
21	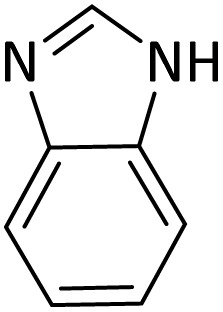	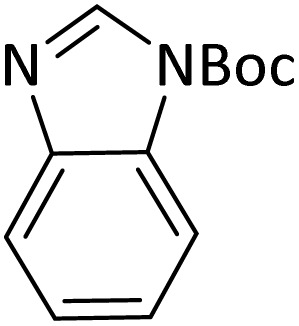	4	94
22	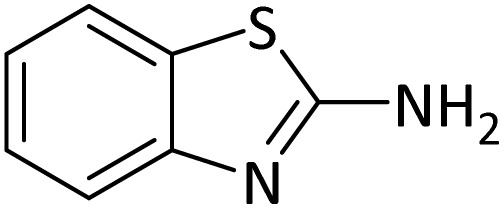	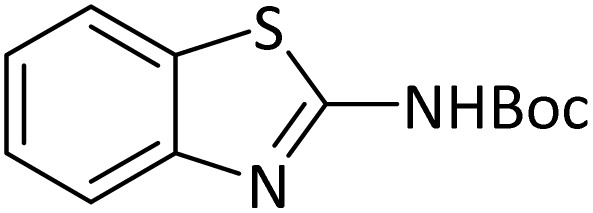	13	95
23	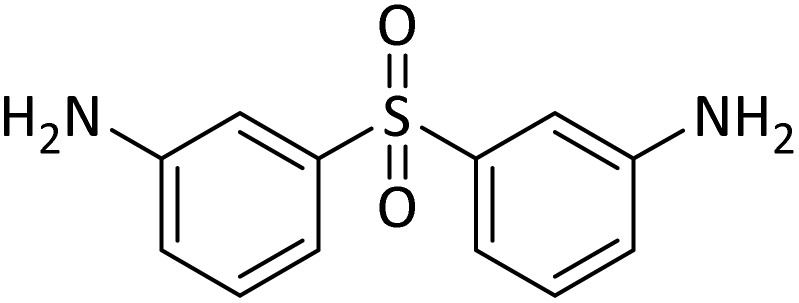	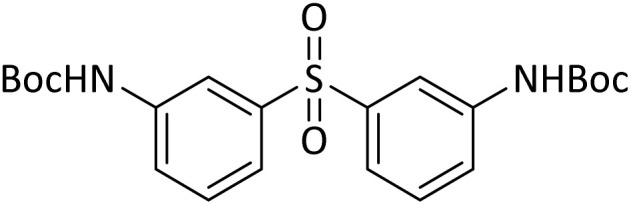	80	95^b^^,^^c^

a Isolated yield.

b 4 mmol of di-*tert*-butoxypyrocarbonate [(Boc)_2_O] was used.

c New product.

According to Table 4, aromatic amines including electron-releasing and electron-withdrawing substituents were reacted successfully and the related *N*-Boc products were obtained in superb yields (Table 4, entries 1–10). Primary and secondary aliphatic amines also resulted in the *tert*-butoxycarbonylated derivatives in 90–96% yields (Table 4, entries 11–16). PINZS worked well in converting aromatic amines with two amine functional groups to the desired products in short reaction times (Table 4, entries 17–19). Superlative chemo selectivity was observed in conversion of amines containing OH functional group so that no *O-tert* butoxy carbonylation be accomplished as side reaction and *N*-Boc product was the major derivative (Table 4, entry 20). The process could also efficiently work with 2-aminobenzothiazole and benzimidazole as heterocyclic amines (Table 4, entries 21–22). The acid sensitive moieties containing methoxy group was being safe in this mild protocol (Table 4, entries 5, 8, 15).

**Scheme 5:**
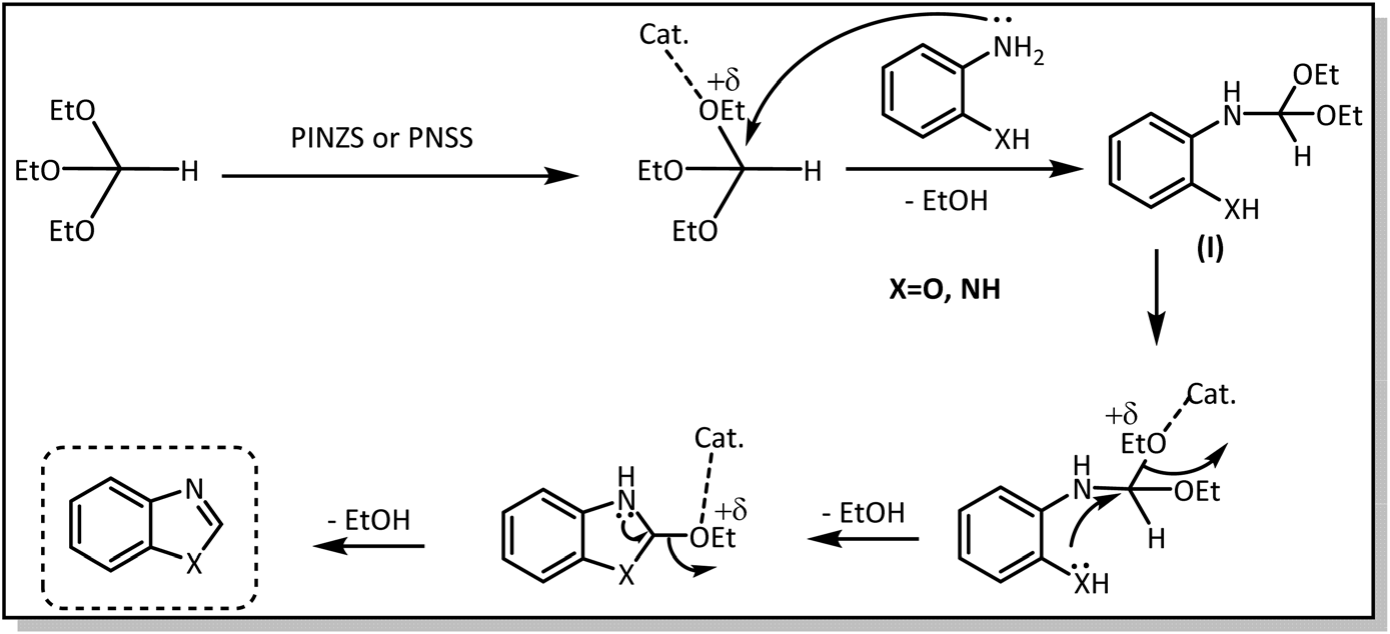
The proposed mechanism for the synthesis of benzoxazoles and benzimidazoles.

The proposed mechanism for the synthesis of benzoxazoles and benzimidazoles in the presence of the introduced catalysts is presented in Scheme 5. According to this mechanism, at first, the catalyst prepares the orthoester through binding to the OEt-group ready for the nucleophilic attack of the amine leading to the intermediate (I) with the release of one H_2_O molecule. Then, the second amino and/or hydroxyl group in this intermediate attack the same carbon center from the other end and releases an ethanol molecule, causing the ring closure. At the end, the third molecule of ethanol is removed and the desired benzoxazoles or benzimidazole is obtained.

**Scheme 6:**
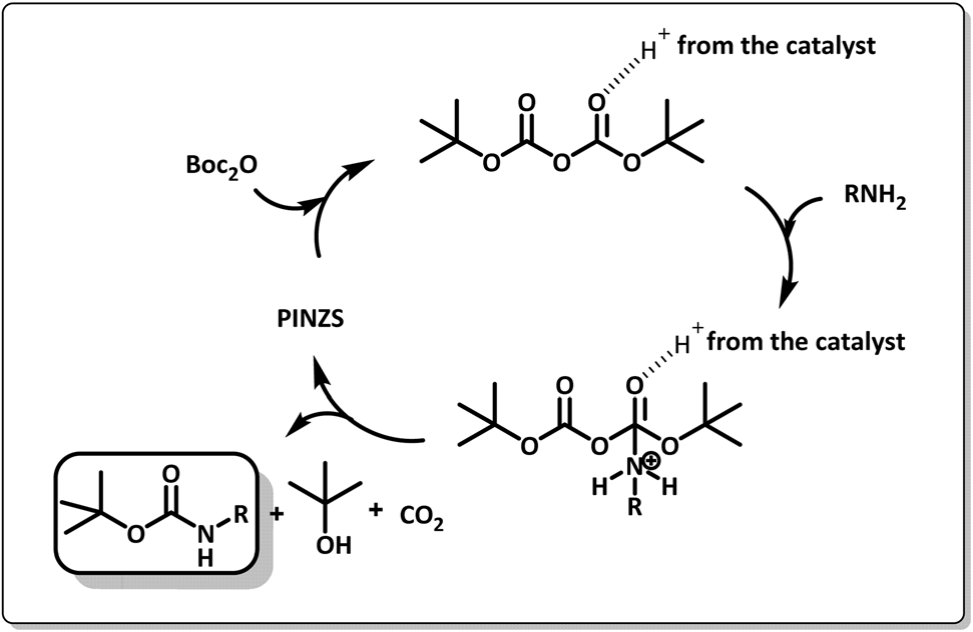
The mechanistic pathway for the *N-tert*-butoxycarbonylation of amines in the presence of PINZS.

The suggested mechanism for the synthesis of amine carbamates by the supporting role of PINZS as the catalyst is shown in Scheme 6. As can be seen, the initial activation of the carbonyl oxygen atoms of (Boc)_2_O is possible by the catalyst. In the next step, after the nucleophilic attack of the amine to carbonyl group accompanied by the production of *tert*-butanol and carbon dioxide, the *N*-Boc protected amine was obtained.

In order to ascertain the performance of PINZS as the catalyst, a comparison of this method for the *N*-Boc protection of aniline with some of those reported in the literature is summarized in Table 5. As can be deduced, the present procedure interestingly can resolve some disadvantages in terms of long reaction times (Table 5, entries 1, 2, 3, 7, 8), reusability of catalyst (Table 5, entries 5, 6, 9), moisture sensitivity of the catalyst (Table 5, entries 4), using of volatile organic solvents (Table 5, entries 1, 2, 3, 5) and difficult work-up (Table 5, entries 5, 6). Accordingly, our catalytic system surpassed the previously reported methods.

**Table 5:** Comparison of the obtained results for the *N-tert*-butoxycarbonylation of aniline with some of those reported in the literature

Entry	Catalyst^ref.^	Condition	Time (min)	Yield (%)
1	Yttria-zirconia^55^	CH_3_CN/r.t.	14 h	90
2	Zn(ClO_4_)_2_·6H_2_O^56^	CH_2_Cl_2_/r.t.	12 h	92
3	[H_2_cryptand222](Br_3_)_2_ (ref. 57)	CH3CN/r.t.	5 h	80
4	[Py][OTf]^58^	Solvent free/r.t.	25	95
5	Thiourea^59^	Toluene/60–70 °C	40	95
6	Thioglcoluril^60^	EtOH/40 °C	8	95
7	β-Cyclodextrine^61^	H_2_O/r.t.	150	75
8	Saccharin sulfonic acid^62^	*n*-Hexane/r.t.	60	97
9	Iodine^63^	Solvent-free/r.t.	30	95
10	PINZS^present work^	Solvent free/60 °C	5	95

**Fig. 1:**
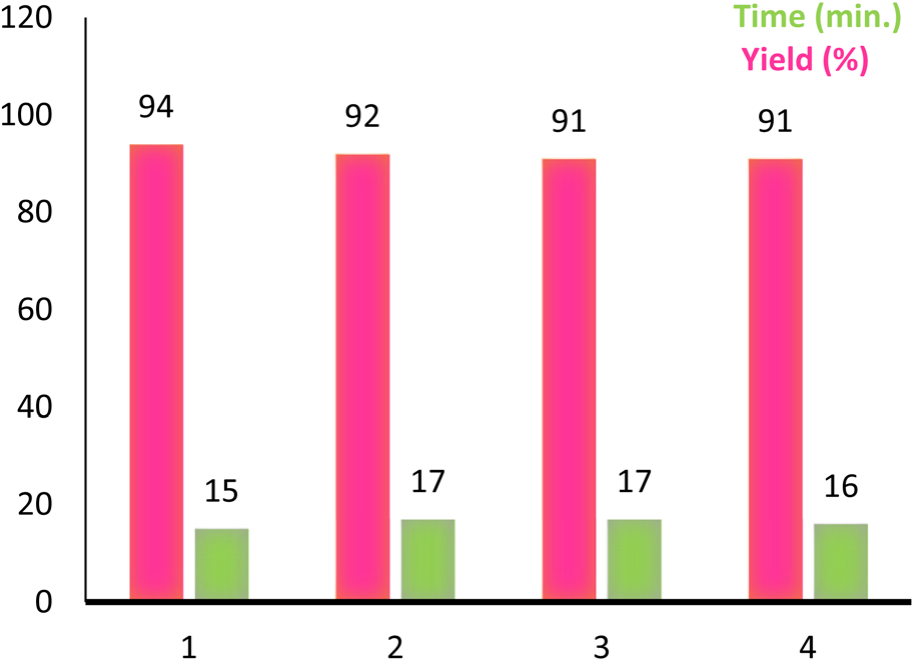
Reusability of PINZS in the *N*-Boc protection of 4-chloroaniline (Table 4, entry 5).

Reusability is an important characteristic which shows the compatibility of a catalyst with the green chemistry rules. This feature for PINZS was confirmed by renewed studying the model reaction (*N*-Boc protection of 4-chloroaniline) under the optimized reaction conditions in the presence of this prepared catalyst. For this purpose, after completion of the reaction, ethanol was added and the catalyst was separated by filtration. The recovered catalyst was washed thoroughly with EtOH, dried and reused for the next run. The observation showed that PINZS is reusable for at least four sequential times and significant changes in terms of the reaction times and yields were not seen (Fig. 1). This topic was proved by comparison of the FT-IR spectra of the recovered catalyst and the freshly prepared one and the figure is presented in ESI.†

## Conclusions

In summary, we presented a comparison of the applications of ZnO NPs and SiO_2_ NPs containing piperazine based ionic liquid as efficacious and high-performance nano catalysts for the acceleration of the synthesis of benzoxazoles and benzimidazoles. The results showed that the target molecules were obtained in shorter reaction times in the presence of ZnO NPs containing catalyst. In the other part, simple and efficient synthesis of the *N*-Boc protected amines was also carried out with more powerful catalyst (PINZS). Several advantages such as solvent-free nature of the reaction, clean and simple operational procedure, short reaction times, high yields and reusability of the catalyst, make this method to be the best route for the protection of amines in organic synthesis rather than the previously reported ones. It should be noted that the selectivity is the significant advantage of this protocol that can be seen in converting of amines containing hydroxyl groups to their *N*-Boc derivatives.

## Conflicts of interest

There are no conflicts to declare.

## Supplementary Material

RA-014-D4RA01300J-s001
