# Supplementary material for: Comparison of the effectiveness of two piperazine based nano-catalysts in the synthesis of benzoxazoles and benzimidazoles and use of the powerful one in the N-Boc protection of amines
Source: RSC Adv. 2024 Mar 19;14(13):9218–27. doi: 10.1039/d4ra01300j (PMC10949119; doi:10.1039/d4ra01300j)
Supplement: RA-014-D4RA01300J-s001 [file RA-014-D4RA01300J-s001.pdf]

Electronic Supplementary Material (ESI) for RSC Advances.
This journal is © The Royal Society of Chemistry 2024

di-tert-Butyl (sulfonylbis(1,3-phenylene))dicarbamate: FT-IR (KBr): v = 3333, 2981, 1730, 1700, 1595, 1533, 1480 cm^{-1}. ^{1}HNMR (500 MHz, DMSO-d_6) σ: 1.59 (9H, s, 3×CH_3), 7.58-7.64 (2H, m, arom), 7.74 (1H, d, *J* =8 Hz, arom), 8.28 (1H, s, arom), 9.92 (1H, s, NH) ^{13}CNMR (100 MHz, DMSO-d_6): 27.97, 79.80, 115.79, 120.55, 122.59, 130.18, 140.67, 141.48, 152.58.

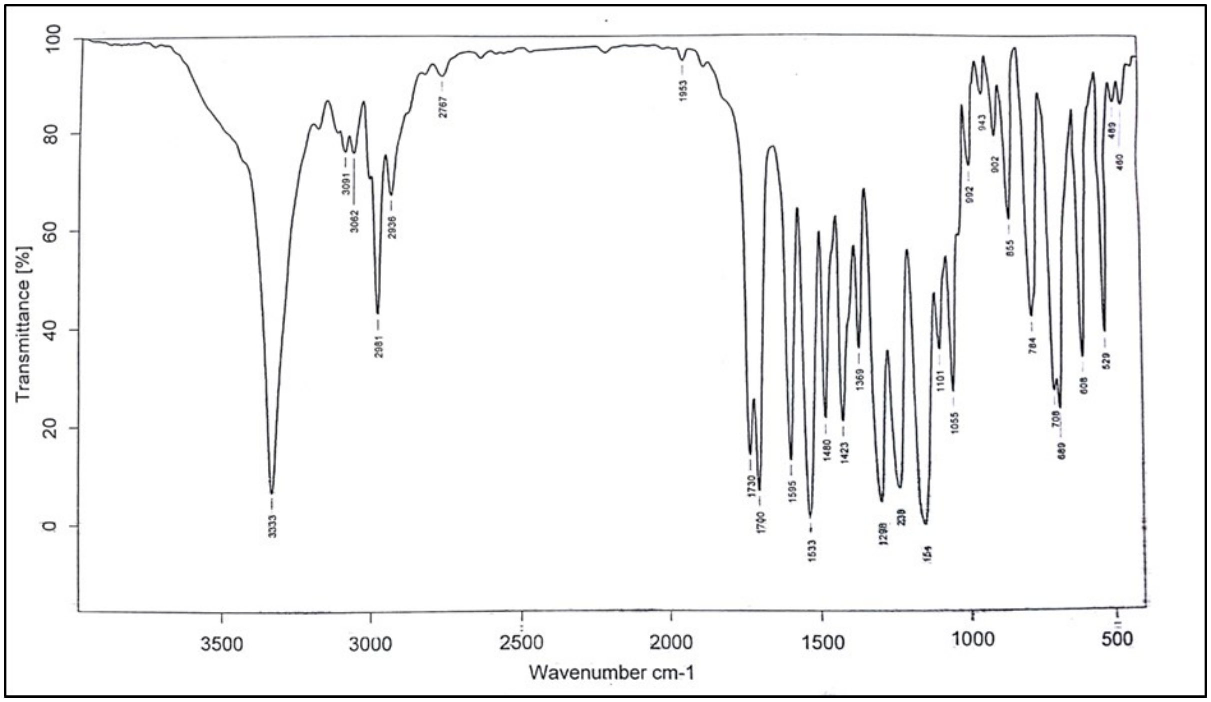

FT-IR spectrum of di-tert-Butyl (sulfonylbis(1,3-phenylene))dicarbamate

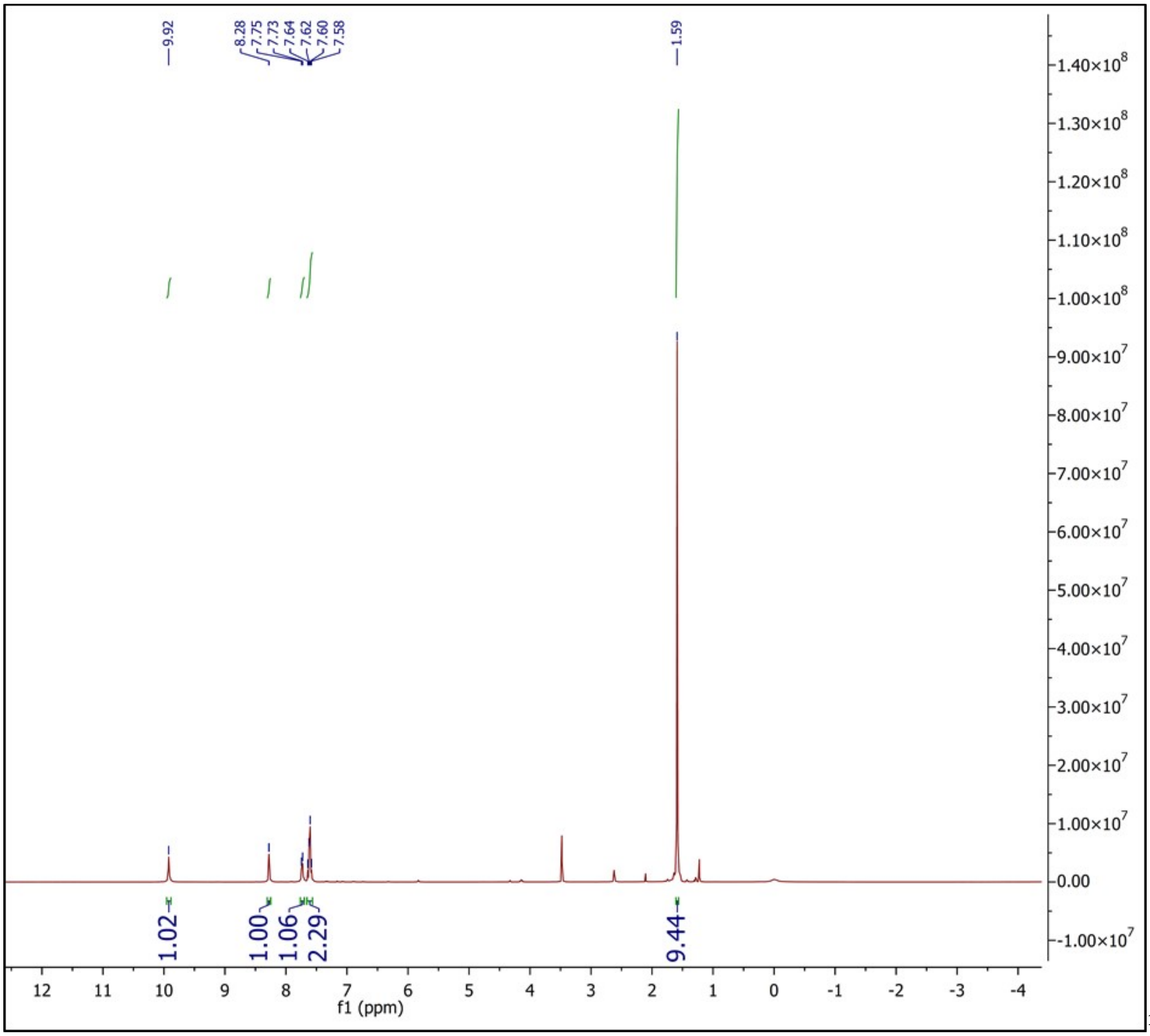

^{1}H NMR spectrum of di-tert-Butyl (sulfonylbis(1,3-phenylene))dicarbamate

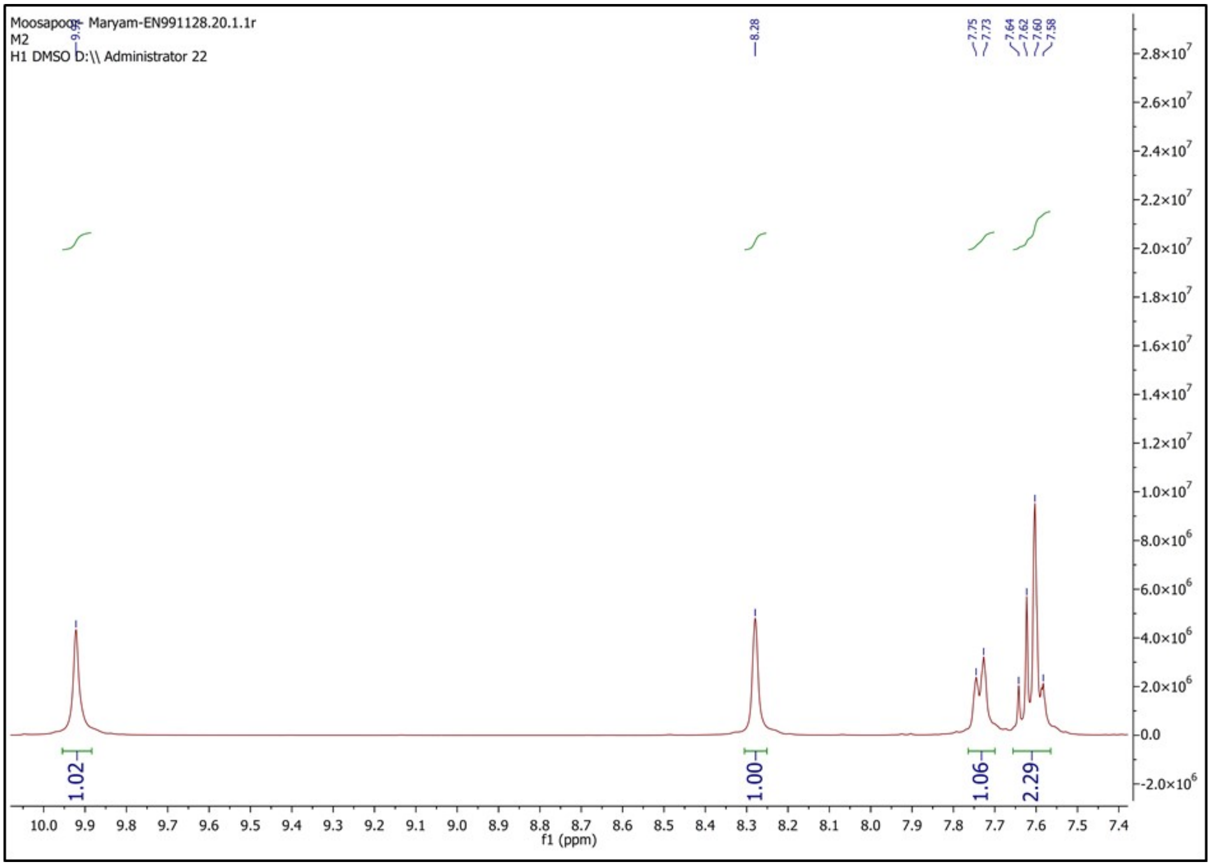

^{1}HNMR spectrum of di-tert-Butyl (sulfonylbis(1,3-phenylene))dicarbamate

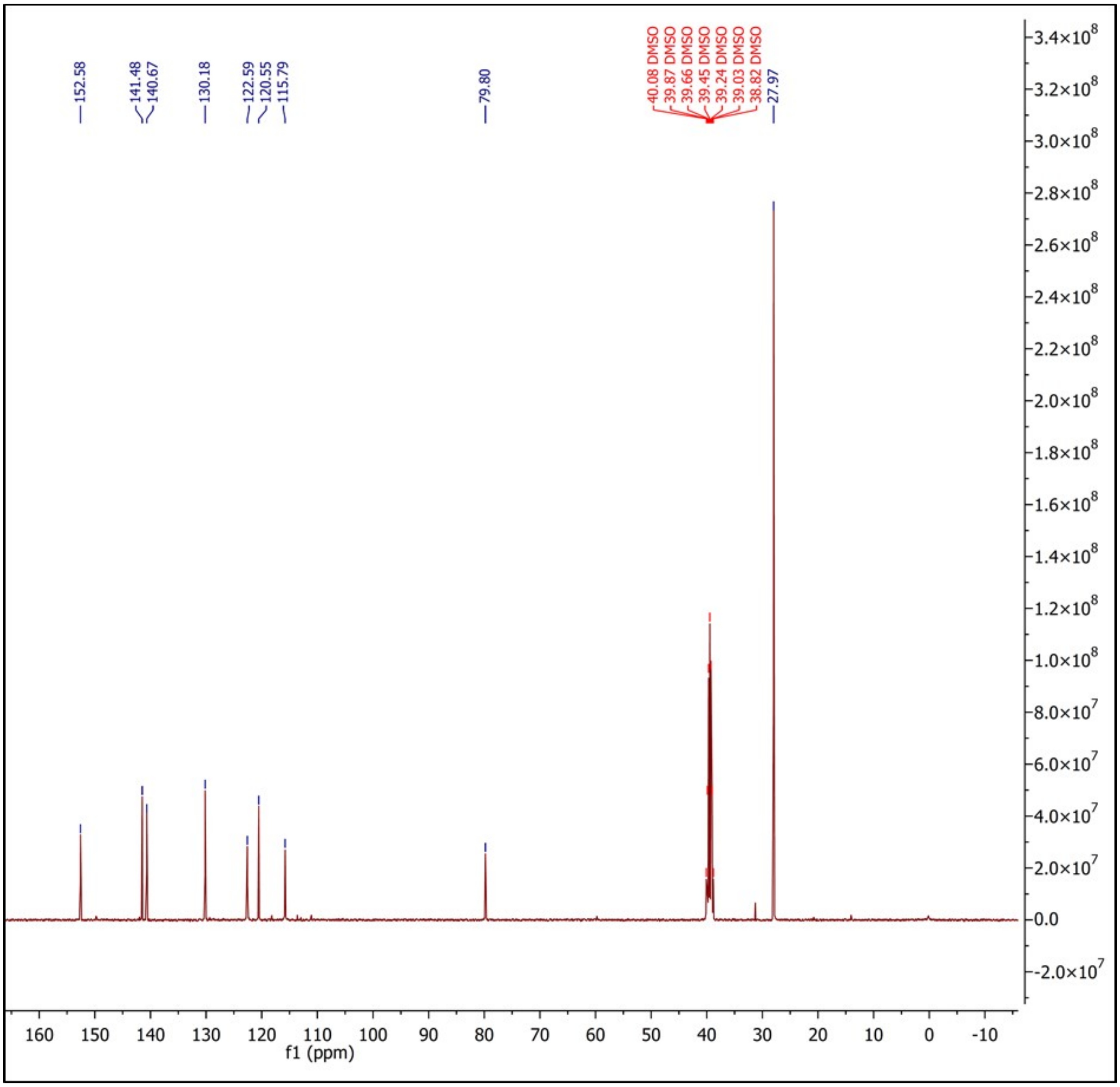

^{13}CNMR spectrum of di-tert-Butyl (sulfonylbis(1,3-phenylene))dicarbamate

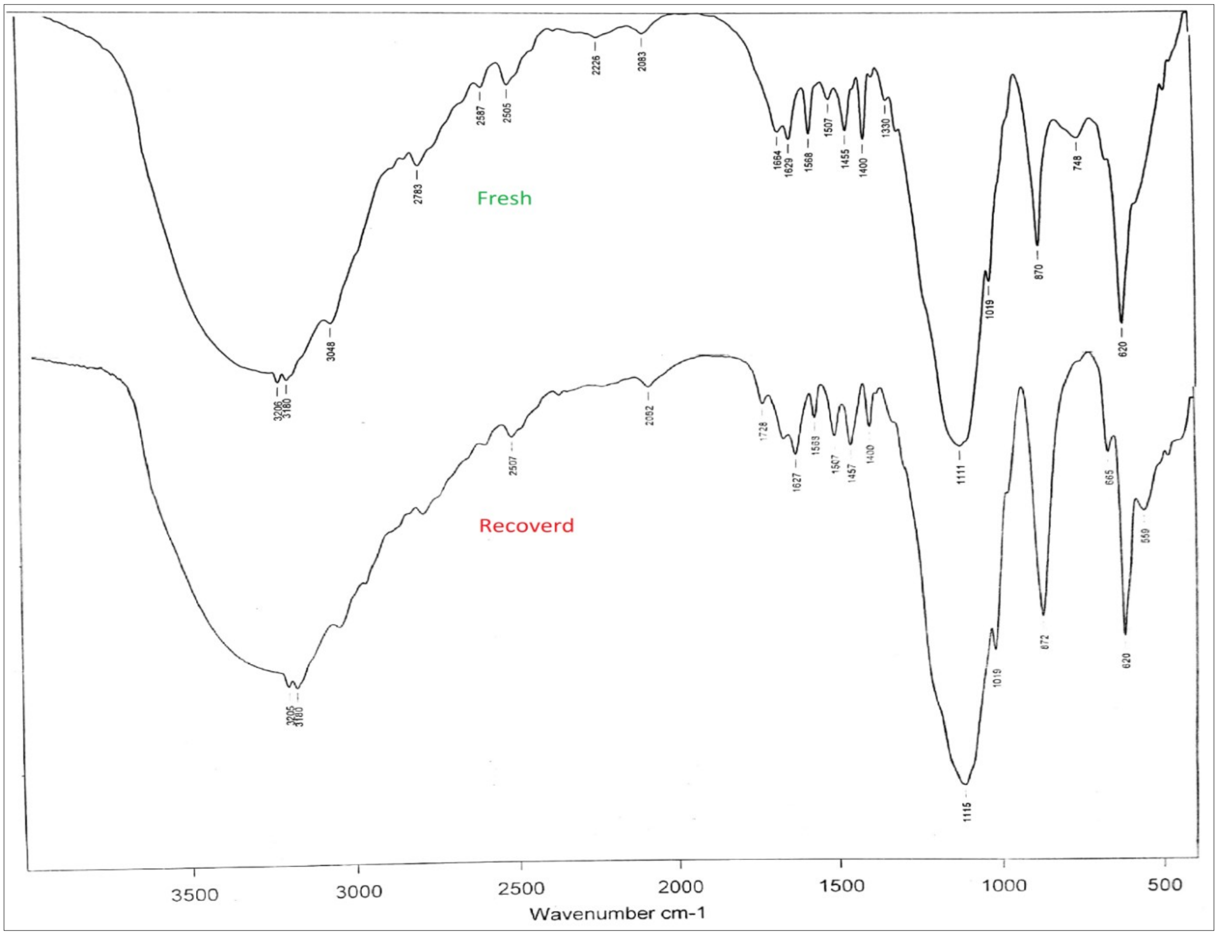

Figure FT-IR spectra of fresh PINZS (up) in comparison with its recovered form (down).
